# Knockdown of c-MYC Controls the Proliferation of Oral Squamous Cell Carcinoma Cells in vitro via Dynamic Regulation of Key Apoptotic Marker Genes

**DOI:** 10.22088/IJMCM.BUMS.10.1.45

**Published:** 2021-05-22

**Authors:** Hussein Sabit, Huseyin Tombuloglu, Emre Cevik, Shaimaa Abdel-Ghany, Engy El-Zawahri, Amr El-Sawy, Sevim Isik, Ebtesam Al-Suhaimi

**Affiliations:** 1 *Department of Genetics, Institute for Research and Medical Consultations, Imam Abdulrahman Bin Faisal University, Dammam, Saudi Arabia.*; 2 *College of Biotechnology, Misr University for Science and Technology, Giza, Egypt.*; 3 *Department of Molecular Biology and Genetics, Faculty of Engineering and Natural Sciences, Uskudar University, Istanbul, Turkey.*; 4 *SANKARA Brain & Biotechnology Research Center, Istanbul Biotechnology Inc, Technocity, Avcilar, Istanbul, Turkey.*; 5 *Department of Biology, College of Science, Imam Abdulrahman Bin Faisal University, Dammam, Saudi Arabia.*

**Keywords:** Oral squamous cell carcinoma, siRNA, c-MYC, knockdown, p27, CYCS

## Abstract

Oral squamous cell carcinoma (OSCC) is the most common malignant epithelial cancer occurring in the oral cavity, where it accounts for nearly 90% of all oral cavity neoplasms. The c-MYC transcription factor plays an important role in the control of programmed cell death, normal-to-malignant cellular transformation, and progression of the cell cycle. However, the role of *c-MYC* in controlling the proliferation of OSCC cells is not well known. In this study, *c-MYC* gene was silenced in OSCC cells (ORL-136T), and molecular and cellular responses were screened. To identify the pathway through which cell death occurred, cytotoxicity, colony formation, western blotting, caspase-3, and RT-qPCR analyzes were performed. Results indicated that knockdown of *c-MYC *has resulted in a significant decrease in the cell viability and c-MYC protein synthesis. Furthermore, caspase-3 was shown to be upregulated leading to apoptosis *via *the intrinsic pathway. In response to *c-MYC* knockdown, eight cell proliferation-associated genes showed variable expression profiles: *c-MYC* (-21.2), *p21 *(-2.5), *CCNA1*(1.8), *BCL*2 (-1.4), *p53*(-3.7), *BAX*(1.1), and *CYCS* (19.3)*. p27* expression was dramatically decreased in *c-MYC*-silenced cells in comparison with control, and this might indicate that the relative absence of *c-MYC* triggered intrinsic apoptosis in OSCC cells *via p27 *and* CYCS*.

Dancer in its broad sense is one of the serious diseases with thousands of deaths worldwide recorded each year (1). Several etiology factors underlie cancer, including bad nutrition habits (2), lack of exercise (3), family history (4), and prolonged exposure to environmental methylation-modulating agents (5-7).

Head and neck squamous cell carcinoma (HNSCC), including oral cancer, is a widespread malignancy with more than 500,000 newly-diagnosed cases per year worldwide (8, 9). Oral cancer is one of the most common malignancies not only in the developing, but also in the developed countries, with more than 405,000 new cases reported each year (10-12). One major type of oral cancer is the squamous cell carcinoma, which is considered the most prevailed histological form accounting for more than 90% of all HNSCC cases (13).

Etiologic factors for oral squamous cell carcinoma (OSCC) include, but are not limited to, alcohol (14) and tobacco (15), where alcohol and tobacco appear to have a synergistic effect in the etiology of OSCC. Other etiologic factors include red meat and salted meat consumption (16), dietary deficiencies (17), and poor oral hygiene (18). These factors cumulatively induce multi-step carcinog-enesis process leading to the accumulation of several genetic (oncogenes and tumor suppressers) and epigenetic (hyper- and/or hypomethylation) mutations (19-21). Recent researches have focused on finding molecular diagnostic/prognostic markers that could help in assigning patients in the right category.

Avian myelocytomatosis virus oncogene cellular homolog (c-MYC) is a member of the MYC family of transcription factors, where it plays an essential role in controlling cell cycle progression (22), programmed cell death (23) and normal-to-malignant cellular transformation (24). Over expression of the c-*MYC *gene is observed in different types of cancer including HNSCC (25).* c-MYC* was also involved in the regulation of telomerase transcription (a major player in the carcinogenesis process) in association with different E26 transformation-specific (Ets) transcription factor family members (26). *p53* (27) and *p16 *(28) are among the critical tumor suppressor genes that are highly studied in OSCC, with *p53* being mutated in about 90% of HNSCC cases (29, 30).

In the present study, it was aimed to determine the role of *c-MYC *in controlling the proliferation of OSCC cells. For this purpose, we silenced the *c-MYC* gene in OSCC cells by means of small interfering RNAs (siRNA). The effects of *c-MYC* inactivation was assessed by cytotoxicity, colony formation and caspase-3 assays. Besides, the expression level of eight cell proliferation-associated genes were quantified by reverse transcription quantitative PCR (RT-qPCR) technique. Western blotting was utilized to confirm the absence of c-MYC protein. The effect of *c-MYC* in controlling the proliferation and death of OSCC was demonstrated for the first time. The results suggest a possible way to control the death of OSCC cells.

## Materials and methods

**Culture of oral squamous cell carcinoma (OSCC) cell lines**

The human oral squamous cell carcinoma cell line (ORL-136(T)) was grown in DMEM media (Gibco-Life Technologies, USA) supplemented with 10% fetal bovine serum (FBS) (Gibco-Life Technologies, USA), hydrocortisone (Sigma-Aldrich, Germany), and antibiotics mix (1% penicillin/streptomycin and 0.1% amphotericin B) (Gibco-Life Technologies, USA). Cells were maintained in an atmosphere containing 95% air and 5% CO_2_ at 37°C.

**siRNA transfection**

siRNA targeting *c-MYC* was purchased from Santa Cruz Biotechnology (USA). The transfection was performed according to the manufacturer's protocol. Briefly, cells were transfected with *c-MYC*-targeting siRNA at final concentration of 50 nM. About 5 × 10^3^ cells were plated in 6-well culture plates for 24 h. All medium were removed, and the plates were washed with transfection medium (Santa Cruz Biotechnology, sc-36868). Solution A was prepared by adding 3 µL duplex siRNA in 50 µL transfection medium while solution B was prepared by adding 3 µL transfection reagent in 50 µL transfection medium. Solution A was added drop wise to solution B and then mixed well. The mixture was incubated for 30 min at 37ºC and then 400 µL of transfection medium was added. The mixture was then overplayed on the cells, with the old medium removed. Plates were incubated for 3 h in CO_2_ incubator. As a negative control, several wells containing OSCC cells were treated with transfection medium and transfection reagent (TM+TR). These cells were subjected to all downstream analyzes parallel to the *c-MYC*-targeting siRNA-treated cells.

**Cell proliferation assay**

Cytotoxicity of siRNA-treated cells was measured using MTT (3-(4,5-dimethylthiazolyl-2)-2,5-diphenyltetrazolium bromide) (Merck, Germ-any) assay according to Angius and Floris (31). Briefly, the harvested cells were re-suspended in 100 μL medium, and were then added to a 96-well microtiter plate (3 × 10^3^ cells/well). Twenty μl of MTT solution (5 mg/mL) was added to each well including control wells. The cells were then incubated for 3 h in CO_2_ incubator. The formed formazan crystals were dissolved by adding 180 μl DMSO (Merck, Germany) to each well. The plate was incubated at room temperature on a shaker at 250 rpm for 30 min, and was read at 545 nm using plate reader (TS Absorbance Reader, BioTek Instruments, USA). All samples were read three independent times, and the average was considered for data analysis. Cells were read after 3, 6, 9, and 12 h. The cell viability was calculated according to the following equation (32): % Cell viability = (OD siRNA-treated / OD control) x 100

**Clonogenic assay**

After transfecting the OSCC cells with *c-MYC*-targeting siRNA for 12 h, the cells were trypsinized and collected by centrifugation at low speed (250 rpm) for 15 min at 4ºC, and then washed twice with PBS. Collected cells were re-suspended in sufficient amount of growth medium, and counted under light microscope. Then, the cells were diluted to a final concentration of 10^4^cells/mL, cultured in 12-well plates and incubated in CO_2 _incubator at 37ºC for at least 7 days or until the colonies started to appear. To stain the cells, the old medium was removed, and the cells were washed twice with PBS (33). Appropriate amount of 6% glutaraldehyde and 0.5% crystal violet were added to each well and left for 45 min at room temperature. The mixture was removed carefully, and the plates were left to dry at room temperature. Formed colonies were counted under light microscope. All experiments were performed in triplicate.

**Caspase-3 assay**

To find the activity of caspase-3 enzyme, a colorimetric Caspase 3 Assay Kit (Sigma-Aldrich, USA) was used according to the recommendations of the manufacturer. The assay was performed in 1 ml reaction mixture, and the absorbance was read by using a plate-reader (Biotek, Neo2) at 405 nm.

**RNA extraction and cDNA synthesis**

Total RNA was extracted from (1) control cells (non-treated), (2) transfection medium + transfection reagent (TM+TR)-treated cells (as negative control), and (3) *c-MYC*-targeting siRNA-treated ORL-136 (T) cells after 12 h of transfection using RNA Isolation System (Qiagen, GmbH, Germany). RNA quality and quantity were checked by using NanoDrop^TM^ 2000 (Thermo, UK) spectrometer. cDNA was synthesized using QIAGEN^®^OneStep RT-PCR kit according to the suggested protocol of the manufacturer (Qiagen, GmbH, Germany).

**Gene expression analysis**

RT-qPCR was used to amplify some marker genes associated with intrinsic apoptotic pathways, oncogenic pathway, and cell cycle control mechanisms (*c-MYC*, *CCNA1*, *p21*, *BCL*-2, *p53*, *BAX*, *p27*, and cytochrome C, somatic (*CYCS*)). Primers sequences are presented in Table 1. About 100 ng of cDNA was mixed with SYBR™ Green PCR Master Mix (Applied Biosystems^TM^, USA), forward (10 pM), and reverse (10 pM) primers. The total volume was brought to 25 µL with molecular biology-grade water. The thermal cycling profile was adjusted as follows: pre-PCR heating for 2 min at 95ºC, then 40 cycles of 94ºC for 45 s, and 56-63ºC (depending on each gene) for 30 s. All reactions were performed in triplicates on StepOnePlus™ Real-Time PCR System (Applied Biosystems, USA). Melting curve analysis was performed in order to determine gene specificity. Actin gene was used as an internal control. 2^-ΔΔCT^ method was employed to calculate the fold changes in gene expression.

**Western blotting**

Proteins were extracted from the OSCC collected from the cell culture. Cells were collected and washed three time with PBS, and incubated with lysis buffer (50 mM Tris/HCl pH 6.8, 2 mM EDTA pH 8.0, 1% SDS, 1% 2-mercaptoethanol, 8% glycerol, and 2% 5 protease inhibitor cocktail) on ice for 30 min. The lysate was spun down for 15 min at 13,000 rpm. Protein concentration was determined by Qubit fluorometer (Invitrogen, USA). 60 μg sample was loaded onto a 15% SDS-PAGE gel, and then the PAGE system was operated at constant voltage (120 V) for 45 min. Then, the proteins were transferred to a nitrocellulose membrane. The membrane was blocked with TBS-T washing buffer (5% skimmed milk dissolved in Tris-buffered saline, and 0.1% Tween -20) and coated with specific primary antibodies in a dilution of 1:1500. Antibodies against c-MYC and *β*-actin were purchased from Santa Cruz Biotechnology (USA). Nitrocellulose membrane was washed and then coated with HRP-conjugated goat anti-rabbit secondary antibody (Amersham Pharmacia, USA). The membrane was washed twice and an enhancing chemiluminescence reagent (Amersham ECL Western Blotting Detection Kit, Amersham Pharmacia, USA) was added (33). The specific protein bands were visualized and photographed.

**Statistical analysis**

At least three replicates of randomized sets were performed for all experiments. Student *t*-test was used to identify the significance. Standard deviations and errors were calculated using Microsoft Excel-based equations. The significant differences were denoted as* P<0.05 and** P<0.01.

**Table 1:** Primers sequences

**Gene **	**Forward 5`- 3`**	**Reverse 5`- 3`**	**Tm (ºC)**	**Size (bp)**
***c-MYC***	CGTCCTCGGATTCTCTGCTC	CTTCGCTTACCAGAGTCGCT	59.8	115
***CCNA1***	TACCTCAAAGCACCACAGCA	TCAAGGAGGCTATGGCAGATTC	59.6	159
***p21***	GACATGTGCACGGAAGGACT	GGGCAGGGTGACAAGAATGT	60.1	677
***BCL-2***	CTGTGGAGCCGGCGAAATAA	CAGGCGTTATCGGTCAGGTT	60.5	100
***TP53***	CGCTTCGAGATGTTCCGAGA	CTGGGACCCAATGAGATGGG	59.8	216
***BAX***	ATGCCCGTTCATCTCAGTCC	GGCGTCCCAAAGTAGGAGAG	58.8	158
***p27***	AAGTGGCTGCATCATTGGGG	CTGGTTTTCGGGATGTTTCTCA	60	556
***CYCS***	TGGGCCAAATCTCCATGGTC	ACACTCCTGATAGTTTGCCACA	60	154
**Actin**	CACCAACTGGGACGACAT	ACAGCCTGGATAGCAACG	60	189

Primer design was performed and validated using Primer BLAST.

## Results

**Fig. 1:**
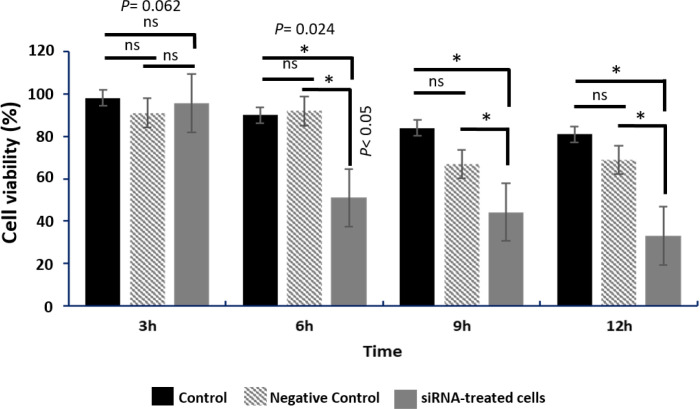
Cytotoxicity assay via MTT. Cells` viability of non-treated (control), siRNA-treated, andtransfection media + transfection reagent (TM+TR)-treated cells (as negative control) were obtained after 3, 6, 9, and 12 h of incubation periods. The significant differences were denoted as * P < 0.05. ns: not significant

**Fig. 2.**
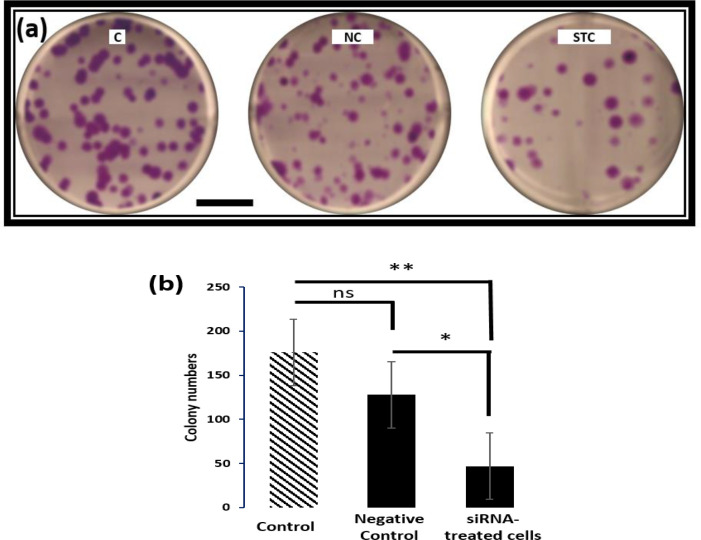
Colonogenic assay. (a) Colonies of OSCC cells after being treated with c-MYC-targeting siRNA. C: control; NC: negative control (TMR+TR); STC: siRNA-treated cells. (b) Significant decrease (**) in the colony count was obtained between control and c-MYC-targeting siRNA-treated cells. The significant differences were denoted with * P < 0.05 and ** P < 0.01. ns: not significant. The scale bar is 4 cm

**Fig. 3.**
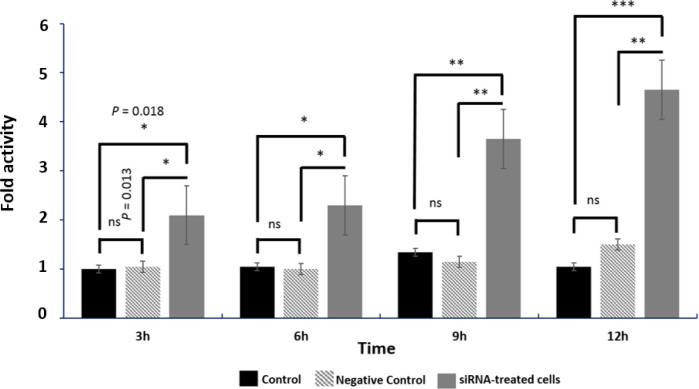
Caspase 3 assay. Primarily, there was a significant increase in the caspase activity in siRNA-treated OSCC cells in comparison with the control. This increase continued until the last incubation period (12 h) where highly significant activity in comparison with the control cells was observed. Significant differences are marked with * P < 0.05 and ** P < 0.1. ns: not significant

In the present investigation, OSCC cells were treated with *c-MYC*-targeting siRNA to determine the actual mechanism by which *c-MYC* induces carcinogenesis. MTT assay was performed on the treated and untreated cells, and the results showed that in the first 3 h of incubation period, no significant differences (P =0.062) were obtained between control and the treatments (Figure 1). Meanwhile, for the 6, 9, and 12 h of incubation periods, the cell viability was significantly changed between siRNA-treated cells and control (P=0.024), and between negative control and siRNA-treated cells (P <0.05). The number of viable cells in siRNA-treated group was significantly decreased in the 12 h incubation period, and the change was increased upon 12 h transfection period. Clonogenic assay was performed to measure the OSCC cell survivability *in vitro* after being treated with *c-MYC*-targeting siRNA (Figure 2). Results indicated a significant decrease (P = 0.04) in the number of colonies formed in the *c-MYC*-targeting siRNA-treated cells in comparison with the control, while no significant difference was detected between control and negative control cells. Caspase 3 assay was performed to identify the cell death pathway initiated by down-regulating *c-MYC* in OSCC cells. Results indicated a significant increase (P = 0.018) in the expression profile of caspase 3 in *c-MYC*-targeting-siRNA-treated cells in comparison with the control and negative control (Figure 3). The induction appeared after 3 h treatment, and gradually increased with increased incubation periods (i.e., 6, 9, and 12 h). This finding revealed that the silencing of *c-MYC* lead to the apoptosis of OSCC cells.

**Fig. 4:**
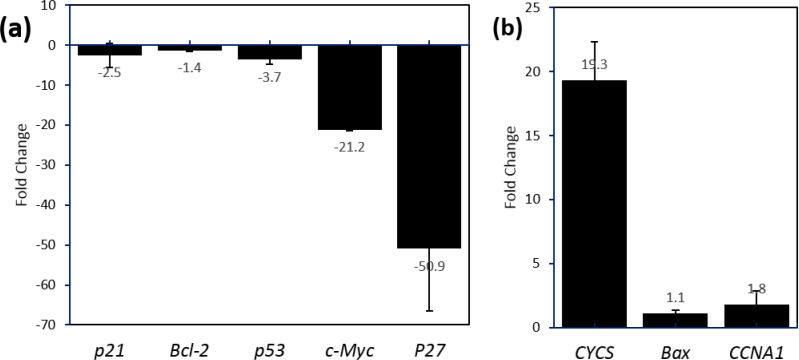
Relative expression of c-MYC, CCNA1, p21, BCL-2, p53, BAX, p27, and CYCS genes. (a) Downregulated and (b) upregulated genes are represented in OSCC cells treated with c-MYC-targeting siRNA in comparison with the control cells. Significant variation in the fold changes was noticed especially in p27 (-50.9), c-MYC (-21.2), and CYCS (19.3).

RT-qPCR was employed to assess the expression level of several cell proliferation-associated genes; *c-MYC*, *CCNA1*, *p21*, *BCL*-2, *p53*, *BAX*, *p27*, and *CYCS*. Results showed a variable fold change in almost all examined genes in comparison with the control (Figure 4). Treating cells with *c-MYC*-targeting-siRNA down-regulated the gene expression of *c-MYC* (21 times), *p21* (2.5 times), *p53* (3.7 times), and *p27 *(51 times); while *CYCS* was up-regulated (19.3 times). The relative fold changes were not significant for *BAX *and *CCNA1*.

**Fig. 5:**
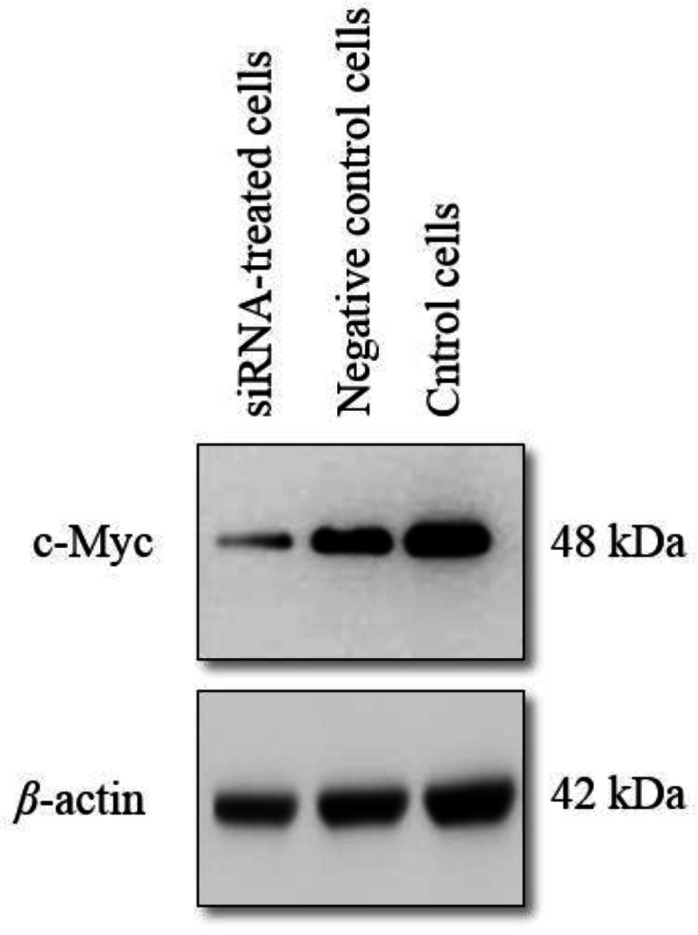
Western blot analysis of expressed c-MYC and β-actin proteins. c-MYC appears to be downregulated in the c-MYC-targeting siRNA-treated OSCC cells in comparison with the control cells

In order to confirm the suppression of *c-MYC* genes, we performed western blot analysis by using c-MYC-specific antibody. Western blotting image is shown in Figure 5. Accordingly, it was shown that *c-MYC* protein was down-regulated in the siRNA-treated cells in comparison with the TM-treated and control cells.

## Discussion

*c-MYC* is constitutively expressed in cancer tissues, which leads in turn, to upregulation of several cancer related genes including, but not limited to, oncogenes (25, 34). In this study, knocking down *c-MYC* in the OSCC cells resulted in significant decrease in the overall cell viability in the siRNA-treated cells as indicated by MTT assay (Figure 1). Clonogenic assay also indicated the suppression of cellular proliferation in cells treated with *c-MYC*-targeting siRNA (35) (Figure 2). Although transient, siRNA-mediated gene knock down is still the best choice to identify the role of such gene in the tumorigenesis and/or tumor spreading (36, 37). 

Furthermore, the results indicated a significant increase (P = 0.018) in the expression of caspase 3 in the *c-MYC*-targeting-siRNA-treated OSCC cells (Figure 3). Because *c-MYC* functions as a regulator of cellular proliferation, its partial absence might lead to different cell death pathways activation, including those involving caspase 3 (38).  This reflects the apoptosis activity. A similar finding was observed in acute lymphoblastic leukemia cells. When c-*MYC* was suppressed by a c-*MYC* inhibitor, the cells underwent caspase-3-dependent apoptosis (39). An inverse relationship between the expression of *c-MYC* and caspase-3 was also observed in this study. However, another study (40) indicated no association between caspase-3 and *c-MYC* expressions, although in non-small cell lung carcinoma cells. Western blotting analysis revealed the partial knock down of *c-MYC* as indicated by the presence of slightly visible protein band (Figure 5). siRNA-mediated downregulation of *c-MYC*, not only induced apoptosis *in vitro*, but also could be used to suppress the growth of OSCC *in vivo *(41).

In the present study, eight genes were subjected to RT-qPCR analysis (*c-MYC*, *CCNA1*, *p21*, *BCL*-2, *p53*, *BAX*, *p27*, and *CYCS*). Data showed that for the cells treated with *c-MYC*-targeting siRNA, a variation occurred in the gene expression profiles, where *CYCS* was upregulated (19.3), while* c-MYC* and *p27* were downregulated (-21.2 and -50.9, respectively). Partial knocking down of *c-MYC* resulted in upregulation of *CYCS*, and this is in contradiction with the finding of Juin et al. (42) who indicated that activation of *c-MYC* triggers the release of CYCS from mitochondria. Iaccarino et al. (43) also indicated that releasing *Cycs* is associated with the activation of *c-Myc* in rats. *c-MYC*, one of the most frequently inordinate oncogenes, is highly expressed in several malignancies including oral cancers. Thus, its inactivation might lead to cell death (44, 45). In this study, knocking down of *c-MYC* have led to *p27* downregulation. Our data are in line with another study (46), which indicated that although activation of *c-MYC* did not result in changes in the expression of *p53*, *p21*^waf1^^/cip1^, *BCL*-2, *B*AX, *B*CL-*xL*, BAD and *cyclins D1*, *E, A and B*, its partial knock down has led to downregulation of *p27,*the potent tumor suppressor gene. Induction of apoptosis by down-regulation of *p27* has been shown in different cell types such as glioblastoma cells (47), mesangial cells and fibroblasts (48). Also, it is found that spontaneous apoptosis in p27-positive tumors is higher than that in p27-negative OSCC (49). In this study, the suppression led to apoptosis as revealed by cytotoxicity assay and western blotting. It is indicated that knocking down of *c-MYC* disrupts the cell cycle control, which, in turn, activates the release of CYCS C and p53 leading to cell death (50), *via* disrupting the *Cdk*/*Rb*/*E2F* pathway, and down regulating *CDK4*, cyclin D1, *CDK2*, *pRb*, *E2F3*, and *DP2* (49, 51, 52). The gene expression level of *p27* is correlated with *c-MYC*, where downregulating* c-MYC *leads to downregulation of *p27* and its stability at protein level (49).

The present study tried to identify the pathway through which OSCC cells committed apoptosis after partial knock down of *c-MYC*. OSCC cells were treated with *c-MYC*-targeting siRNA. Cytotoxicity was measured using MTT assay followed by clonogenic assay and western blotting. The expression of some cancer-related genes (*c-MYC*, *CCNA1*, *p21*, *B*CL-2, *p53*, *B*AX, *p27*, and *CYCS*) was evaluated using RT-qPCR. Results indicated that partial knockdown of *c-MYC* resulted in significant reduction in the cell viability and *c-*MYC protein production. The partial knockdown of *c-MYC* instead of full silencing may have occurred due to off-target effect. Either the sense or antisense siRNA strands may have partial complementarity with non-target mRNAs (53). Caspase 3 assay revealed an intrinsic cell death pathway, where the level of caspase 3 was increased significantly in the partial absence of *c-MYC*. Most of the studied genes were down-regulated in siRNA-treated OSCC cells including, *c-MYC*, *p21*, *p53*, and *p27. *On the other hand, *CYCS* was up-regulated with the knock down of *c-MYC*. These data suggest a possible route for the control of OSCC cells` death. 
